# Decreased Functional Connectivity in Insular Subregions in Depressive Episodes of Bipolar Disorder and Major Depressive Disorder

**DOI:** 10.3389/fnins.2018.00842

**Published:** 2018-11-14

**Authors:** Zhiyang Yin, Miao Chang, Shengnan Wei, Xiaowei Jiang, Yifang Zhou, Lingling Cui, Jing Lv, Fei Wang, Yanqing Tang

**Affiliations:** ^1^Department of Psychiatry, The First Affiliated Hospital of China Medical University, Shenyang, China; ^2^Brain Function Research Section, The First Affiliated Hospital of China Medical University, Shenyang, China; ^3^Department of Radiology, The First Affiliated Hospital of China Medical University, Shenyang, China; ^4^Department of Geriatric Medicine, The First Affiliated Hospital of China Medical University, Shenyang, China

**Keywords:** insula, fMRI, functional connectivity, depressive, bipolar

## Abstract

**Objective:** Clinically, it is very difficult to distinguish between major depressive disorder (MDD) and bipolar disorder (BD) in the period of depression. Increasing evidence shows that the insula plays an important role in depression. We aimed to compare the resting-state functional connectivity (rsFC) of insular subregions in patients with MDD and BD in depressive episodes (BDD), who had never experienced manic or hypomanic episodes when they were scanned to identify biomarkers for the identification of two diseases.

**Methods:** We recruited 21 BDD patients, 40 MDD patients and 70 healthy controls (HC). Resting-state functional magnetic resonance imaging (rs-fMRI) was performed. BDD patients had never had manic or hypomanic episodes when they were scanned, and the diagnoses were determined by follow-up. We divided the insula into three parts including the ventral anterior insular cortex (v-AIN), dorsal anterior insular cortex (d-AIN), and posterior insula (PI). The insular-based rsFC was compared among the three groups, and an analysis of the correlation between the rsFC value and Hamilton depression and anxiety scales was carried out.

**Results:** BDD and MDD patients demonstrated decreased rsFC from the v-AIN to the left superior/middle frontal gyrus compared with the HC group. Versus MDD and HC groups, BDD patients exhibited decreased rsFC from the v-AIN to the area in the left orbital frontal gyrus and left superior temporal gyrus (included temporal pole), from the PI to the right lateral postcentral gyrus and from all three insular subregions to the somatosensory and motor cortex. Meanwhile, a correlation between the rsFC value of the PI-right lateral postcentral gyrus and anxiety score was observed in patients.

**Conclusion:** Our findings show BDD and MDD patients have similar decreases in insular connectivity in the dorsal lateral frontal regions, and BDD patients have specific decreased insular connectivity, especially in the somatosensory and motor cortex, which may be used as imaging evidence for clinical identification.

## Introduction

Bipolar disorder (BD) and major depressive disorder (MDD) belong to different diagnostic categories; however, it is very challenging to clinically identify BD and MDD with only the onset of depression. Although there are some clinical features that could help doctors identify BD early, such as family history (Craddock and Jones, 1999; Taylor et al., 2002; McGuffin et al., 2003), atypical features (Benazzi, 2000), age at onset (Woo et al., 2015), comorbidity with attention deficit and hyperactivity disorder (Torres et al., 2015; Hunt et al., 2016; Usami, 2016), this clinical information cannot be used to reliably and specifically predict the diagnosis. It is still not sufficient for clinicians to quickly diagnose BD with depression. Early research has shown that BD is often misdiagnosed, more than 1/3 of the patients with BD were first diagnosed with depression, and the correct diagnosis took a long time (Hirschfeld et al., 2003). Several studies show that the average annual return rate of MDD diagnosis to BD was approximately 1–2% (Angst et al., 2005; Dudek et al., 2013; James et al., 2015). Among them, a 26-year follow-up study showed that the total diagnostic outcome was 39% (Angst et al., 2005). Misdiagnosis can lead to inappropriate treatment for BD patients, delay the best time to treat, increase the incidence of mania (Hirschfeld et al., 2003) and suicidal behavior, influence the prognosis of disease (Maj et al., 2002; MacKinnon et al., 2005), and increase the economic burden (Peele et al., 2003). Therefore, the early identification of BD with depression and MDD is essential.

Functional magnetic resonance imaging (fMRI) is a promising imaging method to study psychosis both in structure and function. Resting state functional connectivity (rsFC) is a frequently used method which is indicated by the temporal correlations in spatially separated brain regions (Lowe et al., 2000). It may be clinically useful to the identification of BD and MDD in depressive episode (De Almeida and Phillips, 2013; Rive et al., 2016).

Previous MRI studies indicate that BD and MDD show several disrupted brain regions, and the insula could be a key region associated with depression. A recent study on cortical structure from 20 cohorts worldwide, which contained more than two thousand MDD patients, suggest that adults with MDD had thinner cortical gray matter in the insula than controls (Schmaal et al., 2017). Insular regional homogeneity is decreased in MDD and treatment-resistant depression (Yao et al., 2009; Guo et al., 2011). There is also increased insular amplitude of low frequency fluctuation in treatment-free MDD patients (Liu et al., 2014). Cognitive task research displays that differential activation of the anterior/middle insula was significantly reduced in adolescents with MDD facing expressions of sadness and happiness compared to controls and correlated negatively with depression severity (Henje Blom et al., 2015). Meta-analyses and reviews also reported altered insular volumes in BD patient (Ellison-Wright and Bullmore, 2010; Dell’Osso et al., 2014), and found the anterior insular volume was inversely correlated with the lifetime number of depressive episodes (Takahashi et al., 2010). Depressive episodes of BD have been associated with increased serotonin transporter binding in the insula using positron emission tomography (Cannon et al., 2007). Studies also found the abnormal activation of the insular cortex in unmedicated BD patients and patients with bipolar depression (Liu et al., 2012; Yip et al., 2014). Reviews and meta-analyses for the first-degree relatives of BD patient report that the genetic liability for BD could be related to gray matter abnormalities in insular cortex (Nery et al., 2013; Lee et al., 2014).

The insula is a functionally and cytoarchitectonically diverse region of cortex located deep inside the lateral sulcus of the Sylvian fissure (Ture et al., 1999). The insula connects to several cortical regions with different neural circuits and is involved in multiple functions, including emotion, cognition, sensory perception, and somatosensation (Augustine, 1996; Craig, 2002, 2009; Nieuwenhuys, 2012). Based on the previous structural and functional studies, the insula is considered to have three different subregions, the ventral anterior insular cortex (v-AIN), dorsal anterior insular cortex (d-AIN), and posterior insula (PI) (Mesulam and Mufson, 1982a,b; Deen et al., 2011; Jakab et al., 2012). The AIN affects emotional and cognitive function more, while the PI is more associated with motor-related functions and sensory perception (Mutschler et al., 2009; Kurth et al., 2010; Kelly et al., 2012). Insular subregions exhibit different functional patterns to distinguish diseases.

In the current study, we used a seed-based fMRI method to examine the shared and distinct rsFC between participants with MDD and BD in depressive episodes (BDD). BDD patients had never had mania or hypomania episodes when they were scanned. Few studies have used insular subregion rsFC to study the difference between BDD and MDD, and the sample of BDD patients cannot exclude the effect of mania or hypomania on the brain. The v-AIN, d-AIN and PI were selected as the regions of interest (ROIs), and we explored rsFC between these seed regions to other brain regions. We hypothesized that both the MDD and BDD groups would demonstrate altered rsFC when compared to the control group; furthermore, we hypothesized that there would also be distinct changes in insular rsFC in MDD and BDD.

## Materials and Methods

### Participants

Twenty-one BDD patients, forty MDD patients and seventy healthy controls (HC) participated in the study. Participants ranged from 16 to 48 years old. HC participants were recruited via advertisements. Patients were recruited from two hospitals, the First Affiliated Hospital of China Medical University and the Shenyang Mental Health Center. At least two psychiatrists diagnosed the patients, respectively and came to the same diagnosis. All patients in this study met the Diagnostic and Statistical Manual of Mental Disorders-IV (DSM-IV) criteria for MDD or BD but without other DSM-IV Axis I diagnosis. HC participants had no current or lifetime mental disorders and no history of mental disorder in first-degree relatives (determined by a psychiatrist). Symptoms were measured using the 17-item Hamilton Depression Scale (HAMD-17), Hamilton Anxiety Scale (HAMA) and Young Mania Rating Scale (YMRS). Four sub-scores of the HAMD-17 scale were calculated based on the previous study (Pancheri et al., 2002). The first factor was somatic anxiety saturated by somatic anxiety, hypochondriasis, general somatic symptoms, gastrointestinal symptoms and scale items concerned with insomnia. The second factor was psychic anxiety defined by psychic anxiety, agitation, feelings of guilt and loss of insight. The third factor was a depressive dimension with high loadings on depressed mood, work and interests, and retardation. The fourth factor was anorexia included items such as weight loss and gastrointestinal symptoms.

Participants were excluded if they met any of the exclusion criteria as follows: (1) MRI contraindications; (2) any major medical diseases (hypertension, diabetes, heart disease, liver and kidney diseases, blood diseases or metastatic disease); (3) any neurological disorder or head trauma with 5 min or more loss of consciousness; (4) any somatic diseases with psychotic symptoms (multiple sclerosis, typhoid, thyroid diseases); (5) any kinds of personality disorders; autism or developmental disorder; (6) obvious substance abuse/dependence within 3 months before recruitment; (7) pregnancy; or (8) IQ<70.

Follow-up: All patients were followed up by telephone every three months after participation in the study. The forty MDD patients were followed up for more than 12 months without a change in diagnosis. The twenty-one BDD patients were in depressive episodes in the scan with the diagnosis of MDD. At the time of enrollment, we asked in detail whether there had been a history of manic or hypomanic episodes that met the DSM-IV criteria. During the follow-up period, if mania or hypomania is found, patients were suggested to see the psychiatrists again as soon as possible. When patients confirm that mania or hypomania is first appeared during the reassessment, they will be included in the BDD group of this study.

All participants were told of the content of the current study. Participants signed informed consent before the study process began. If the participant was underage, the guardian would sign the agreement.

### Data Acquisition

MRI scans of all participants were carried out after recruitment using a GE (General Electric, Milwaukee, United States) Signa HDX 3.0T scanner with a standard 8-channel head coil. In the scanning process, the head of participant was fixed with a foam pad and the participants were kept in a state of rest: lying on their back, eyes closed (as much as possible to keep the eyeballs fixed) and trying not to think. Resting state fMRI scanning with a T2-gradient echo planar imaging (EPI-GRE) sequence: repetition time (TR) = 2000 ms, echo time (TE) = 30 ms, flip angle = 90°, field of view (FOV) = 240 mm^2^ × 240 mm^2^, matrix = 64 × 64, voxel size = 3.0 mm^3^, 35 contiguous slices of 3 mm without gaps, scanning time 6 min 40 s with 200 volumetric images per participant. For the image calibration and participant screening, high-resolution images were scanned with a three-dimensional fast spoiled gradient-echo (FSPGR) sequence. The parameters were as followed: TR/TE = 7.1/3.2 ms, FOV = 240 mm^2^ × 240 mm^2^, matrix = 240 × 240, voxel size = 1.0 mm^3^, 176 contiguous slices of 1 mm without gaps, scanning time 8 min 22 s.

### Data Processing and Analysis

#### fMRI Data Preprocessing

Preprocessing of magnetic resonance imaging data and measurement calculations were managed by the DPABI (Yan et al., 2016) package based on MATLAB 2011a using the following validated steps: converting the DICOM into the NIFTI format; removing the first 10 time points; slice timing; realignment; nuisance covariates, including the Friston 24 motion model, white matter signal, and cerebrospinal fluid signal, and global signals; spatial normalization to the standard Montreal Neurological Institute (MNI) space and resampling into 3 mm^3^ × 3 mm^3^ × 3 mm^3^ voxels; smoothing by a Gaussian filter of 6-mm full-width at half-maximum (FWHM); and filtering low frequency signal by 0.01–0.08 Hz. All functional dates would be checked: if a head motion parameter more than 2.5 mm in displacement or 2.5° in rotation, the subject was excluded from the final analysis.

#### ROI Definition

The insular subregions were subdivided based on a data-driven functional connectivity study (Deen et al., 2011). Specifically, connectivity maps were computed for each voxel within the left and right insula from resting-state data. Then, the insula was parcellated based on connectivity maps using cluster analysis. Finally, three insular subregions with distinct connectivity patterns were identified, including ventral and dorsal anterior regions, and the posterior region. In this study, the three insular subregions were defined as the ROIs.

#### Seed-Based rsFC Calculation

We calculated the mean time courses of the voxels in the insular ROIs for each participant. A cerebral gray matter template including 90 labels of the Anatomical Automatic Labeling (AAL) template was created. Pearson Correlation was performed between the insular ROIs and other cerebral gray matter regions at voxel level. The correlation coefficients were converted to *z*-values using the Fisher r-to-z transformation.

### Statistical Analysis

Demographic and clinical data were analyzed using chi-squared test (for gender), independent sample *t*-test (for clinical scales) and non-parametric test (for age) with a significant threshold of *p* < 0.05. The statistical software was SPSS version 22.0.

The rsFC results of the three groups were analyzed by ANOVA using DPABI software. Monte Carlo simulation (AlphaSim) correction for multiple comparisons was carried out with the significant threshold set at *p* < 0.005 in a voxel-level and *p* < 0.05 in a cluster level. The rsFC values were extracted from the significant ANOVA results of all groups. Then, we performed *post hoc* comparisons between groups for significant regions with Bonferroni correction (Statistical significance was set at *p* < 0.05).

The correlation analyses between HAMD-17 and HAMA scores and rsFC values in the significant regions was performed.

## Results

### Demographic and Clinical Data

Statistical results showed that the three groups were matched in gender. Age of HC and MDD groups did not conform to normal distribution. we made a non-parametric test (Kruskal-Wallis test) and found that there was no statistically significant difference in age among the three groups. HAMD-17 and HAMA scores of the patient groups were significantly different from the HC group, but there was no statistically significant difference between patient groups. The data of demographic and clinical scales are displayed in Table 1.

**Table 1:** Demographic and clinical data of participants.

Characteristics	BDD	MDD	HC	*T/χ^2^*	*P*-value
					
	*N* = 21	*N* = 40	*N* = 70		
Gender (male/female)	10/11	15/25	31/39	0.721	0.697
Age (Mean ± SD)	29.29 ± 8.35	29.55 ± 10.11	29.39 ± 8.082	0.006	0.723
First-episode (Y/N)	18/3	37/3	NA	NA	NA
Antidepressant drugs (Y/N)	9/12	21/19	NA	NA	NA
HAMD-17 total score	22.70 ± 9.056	22.36 ± 9.596	0.89 ± 1.432	-0.12	0.896
HAMA total score	15.76 ± 10.704	17.51 ± 10.294	0.66 ± 1.268	0.568	0.573
YMRS total score	0.941 ± 1.887	0.833 ± 1.621	0.175 ± 0.658	NA	NA

*SD, standard deviation; p < 0.05 was considered statistically significant. Number of participants: HAMD-17 (BDD = 20, MDD = 40, HC = 60); HAMA (BDD = 17, MDD = 35, HC = 59); YMRS (BDD = 17, MDD = 30, HC = 57).*

### ANOVA Results for Three-Group Comparisons

**FIGURE 1:**
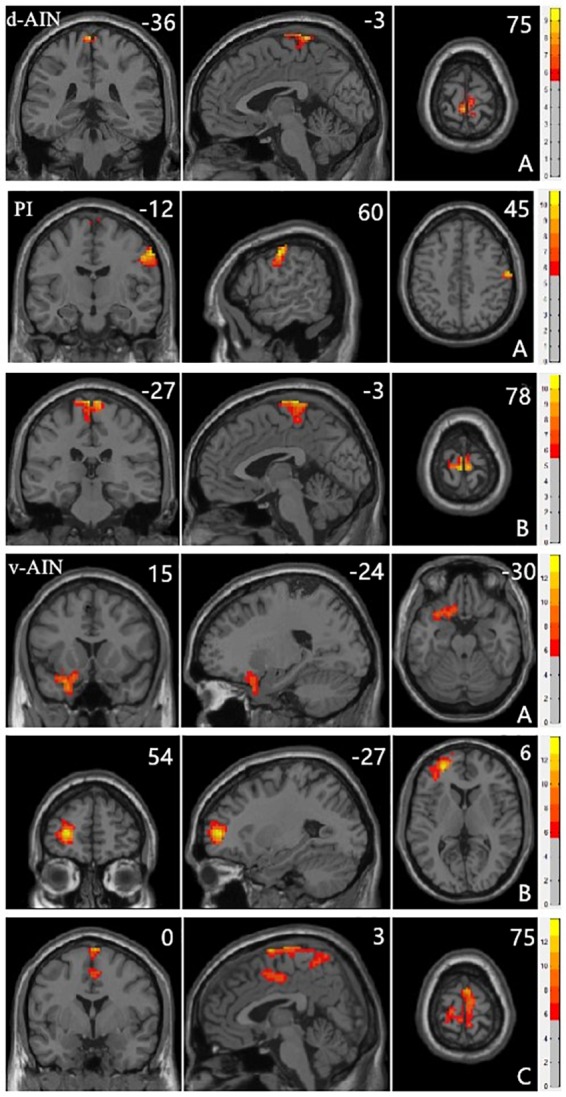
Clusters showing significant differences in the three-group analysis. Regions listed in Table 2. The color bar represents the range of *F* values. The number represents coordinates for each slice.

All significant results reported were corrected (Alphasim, *p* < 0.05) and results of head motion as a covariate showed in Supplementary Material. For the d-AIN connectivity, significant group differences were observed in the bilateral paracentral lobule, precentral gyrus and postcentral gyrus; for the v-AIN connectivity, significant regions contained the left orbital frontal gyrus, left lateral frontal gyrus, left superior temporal regions, paracentral lobule, supplemental motor area, precuneus, precentral gyrus, postcentral gyrus and middle cingulum; for the PI connectivity, one significant region was overlapped with d-AIN, the other region was the right postcentral gyrus. The results are showed in Figure 1 and Table 2.

**Table 2:** Brain regions showing significant differences in the three-group analysis.

Significant regions	Cluster size	Peak MNI coordinate			*F* value
		X	Y	Z	
**Dorsal anterior insular**					
A. Paracentral lobule	93	-3	-36	75	8.4837
Precentral gyrus					
Postcentral gyrus					
**Ventral anterior insular**					
A. Left inferior orbital frontal gyrus	147	-24	15	-30	10.2982
Left superior orbital frontal gyrus					
Left superior temporal pole					
Left superior temporal gyrus					
B. Left middle frontal gyrus	257	-27	54	6	13.9032
Left superior frontal gyrus					
C. Paracentral lobule	822	3	0	75	12.0893
Supplementary motor area					
Precentral gyrus					
Postcentral gyrus					
Precuneus					
Middle cingulum_					
**Posterior insular**					
A. Right postcentral gyrus	88	60	-12	45	10.9103
B. Paracentral lobule	186	-3	-27	78	10.1173
Precentral gyrus					
Postcentral gyrus					

*MNI, montreal neurological institute; X, Y, and Z are MNI coordinates referring to the center of gravity of the cluster.*

### *Post hoc* Analysis

**FIGURE 2:**
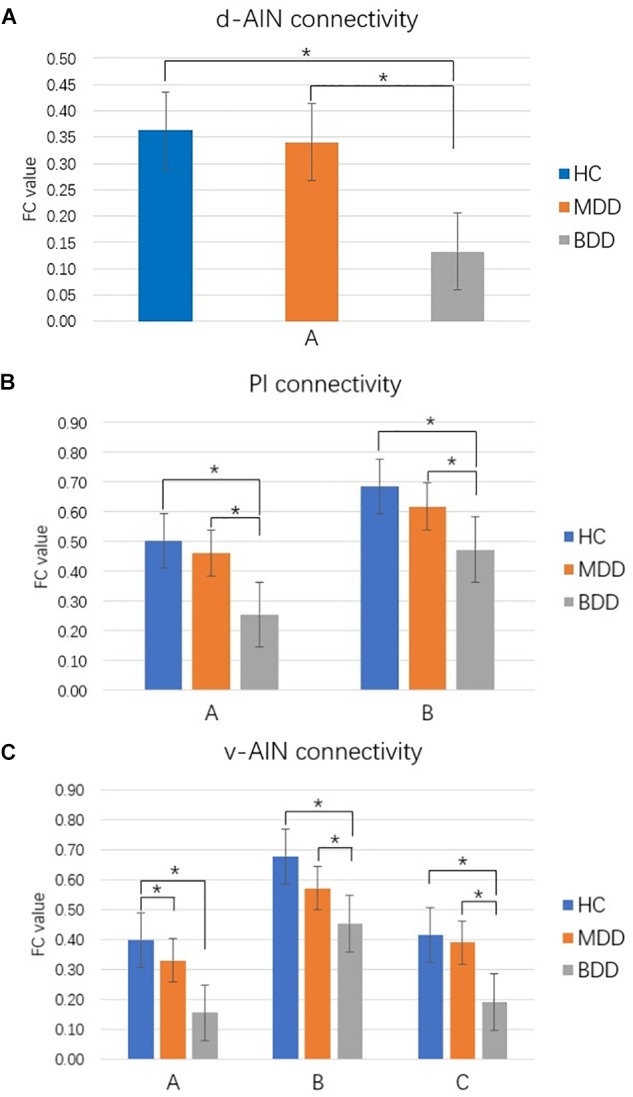
Comparison between groups for significant brain regions. ^∗^*p* < 0.05 Bonferroni corrected. **(A)** d-AIN functional connectivity. **(B)** PI functional connectivity. **(C)** v-AIN functional connectivity. The regions listed in Table 2.

Comparison between each pair of groups of the significant brain regions was carried out. Correction for multiple comparisons was performed by Bonferroni correction at the significant threshold of *p* < 0.05. Decreased rsFC between d-AIN/PI and paracentral lobule, precentral gyrus and postcentral gyrus was observed in the BDD group compared with MDD and HC groups; for the v-AIN, the rsFC decreased areas overlapped but were more extensive and included the paracentral lobule, supplemental motor area, precuneus, precentral gyrus, postcentral gyrus, and middle cingulum. Meanwhile, BDD group exhibited decreased rsFC between the PI and right lateral postcentral gyrus, and between the v-AIN and left superior frontal gyrus (SFG)/left middle frontal gyrus (MFG) versus MDD and HC. In addition, MDD and HC group indicated no significant difference in above rsFC. Decreased rsFC between v-AIN and the area of the left orbital frontal gyrus and left superior temporal gyrus (including the temporal pole) was observed in both the BDD and MDD groups compared with HC group. BDD and MDD patients exhibited no statistical difference with Bonferroni correction *(p* = 0.056), but the BDD demonstrated a lower trend compared to MDD. These results are showed in Figure 2.

### Correlation of Variables

For the MDD group, a negative correlation was reported between the rsFC value of the PI-right postcentral gyrus and HAMA total score (*R* = -0.386, *P* = 0.042). For the BDD group, a positive correlation was observed between the rsFC value of the PI-right postcentral gyrus and the HAMD-17 sub-score of somatic anxiety (*R* = 0.458, *P* = 0.042).

## Discussion

In this study, we compared the insular subregions rsFC with whole brain among BDD, MDD, and HC. The choice of BDD patients greatly eliminated the influence of mania or hypomania on brain function. We found that significant changes mainly exist with the frontal and parietal lobes. The PI and d-AIN aberrant rsFC were observed mainly in the parietal lobe, but the v-AIN aberrant rsFC involved a wider range of regions included both the prefrontal lobe, parietal lobes and a small part of the temporal lobe. The common change of the BDD and MDD groups was the decreased rsFC of v-AIN with the left SFG and MFG. We did not find specific changes in the MDD group compared to the other two groups. For the BDD group, specific decreased rsFC included the primary somatosensory cortex with PI and d-AIN, right postcentral gyrus with PI, motor cortex with v-AIN and left orbital frontal gyrus and left superior temporal area with v-AIN.

The current study found a decreased rsFC of the left dorsolateral SFG and MFG with v-AIN in both BDD and MDD patients. These two aberrant regions of dorsolateral prefrontal cortex (DLPFC) were consistent with previous studies. Previous studies reported that first-episode and medication-naive MDD patients had altered gray matter volume in SFG and MFG, which was negatively correlated with the duration of illness and the severity of clinical symptoms (Lai and Wu, 2014; Peng et al., 2016). Functional studies reported abnormal activation in the SFG, which was associated with rumination in MDD (Guo et al., 2013; Schiller et al., 2013). Task-related and resting state functional studies reported the abnormal activation in the MFG (Naismith et al., 2010; Liu et al., 2013), which was associated with anticipatory emotional processes and depressive symptoms such as apathy, anergia and loss of motivation in MDD (Feeser et al., 2013). Resting state functional studies also revealed abnormal regional homogeneity in the MFG on BD in depressive episodes (Liang et al., 2013; Gao et al., 2014). From these studies and our results, we considered that altered rsFC of DLPFC with v-AIN was the common pathophysiological basis of MDD and BDD. In addition, we found that there was a lower trend of decreased rsFC in BDD. One structural MRI study also found that depressed BD patients showed reduced thickness in the DLPFC compared to MDD (Lan et al., 2014). We surmised that there was more serious dysfunction in BDD.

BDD patients showed specific decreased v-AIN rsFC in left orbital frontal gyrus and left STG (included the left superior temporal pole), which were involved in fronto-limbic neural circuit. The orbital frontal cortex (OFC) participates in the process of learning, predicting and making emotional decisions, as well as the process of rewarding related behaviors (Kringelbach, 2005). Altered function in OFC might be associated with symptoms such as emotional instability and indecisiveness in BDD (Liu et al., 2012). Studies have reported that OFC volume decreases in both adults and children with BD (Nugent et al., 2006; Najt et al., 2007). Recent twin and family fMRI studies of BD also found that OFC rendered the most significant heritability estimates and was significantly correlated with the BD phenotype (Sugihara et al., 2017). These studies indicate that OFC could play an important role in BD. Decreased OFC-insular connectivity was correlated with the processing of emotional information, which leaves the patients with the inability to properly treat their emotions, leading to increased negative self-focus (Critchley et al., 2004; Guo et al., 2015). The superior temporal lobe participates in the processing of emotional experiences. Studies reported that the STG and the adjacent cerebral cortex play important roles in the processing of information related to individual communication (Narumoto et al., 2001). Functional and DTI studies reported the abnormal degree centrality and mean diffusivity of the temporal pole in BD (Zhou et al., 2017; Spuhler et al., 2018). Reduced rsFC between insula and the left STG related areas might result in social withdrawal and be related to the time and duration of the episode (Guo et al., 2015). Together with these results, we considered that the decreased rsFC of vAIN-OFC and vAIN-superior temporal gyrus were specific in the pathophysiology of BDD.

Other significant regions of BDD were in the motor cortex and included the primary somatosensory cortex and supplemental motor area (SMA), which had decreased rsFC with all three insular subregions. The primary somatosensory cortex mainly participates in the processing of sensory information and face emotion (Keysers et al., 2010). Previous studies found that abnormal overactivation by BD depressed patients in response to happy faces and fearful faces (Chen et al., 2006). A study found increased rsFC between the somatosensory cortex and insular cortex in euthymic BD subjects compared to HC (Minuzzi et al., 2017). This was mutually verified by our results. The SMA participants in movement control and is involved in identifying and describing emotional feelings (Han et al., 2018). A study found different activation patterns of SMA in manic and depressed BD during reaction time tasks (Caligiuri et al., 2004). Overall, we speculated that the rsFC of the insula with the primary somatosensory cortex and SMA could be a mood state-related indicator in BD that increases in manic/hypomanic episodes and decreases in depression. The decreased rsFC could distinguish BDD from MDD.

In addition, we found that the rsFC between PI and the right lateral postcentral gyrus was correlated with anxiety in patients. However, the correlation patterns were different. Depression often has anxiety symptoms. The mechanism is still unclear. In this study, we considered that the insula regulates part of the somatosensory cortex and plays an important role. Further research is needed in the future.

The present study has several limitations. First, the sample size of BDD patients was small because the BDD patients were drawn from the initial group of MDD patients after follow-up. Second, we are not sure whether the drug will affect the results. Finally, we did not conduct cognitive tests and we did not carry out classified studies to further verify our results.

## Conclusion

BDD and MDD patients showed similar and distinct decreased insular rsFC changes. BDD patients have specific decreased insular connectivity, especially in the somatosensory and motor cortex, which may be used as imaging evidence for clinical identification.

## Ethics Statement

All proceduresperformed in studies involving human participants were in accordance with the ethical standards of the institutional and/or national research committee and with the 1964 Helsinki Declaration and its later amendments or comparable ethical standards. The study was approved by the Medical Science Research Ethics Committee of the First Affiliated Hospital of China Medical University (approval reference number [2012]25-1). All participants provided written informed consent by themselves or via their parents/guardians if they were under 18 years old after a complete description of the study.

## Author Contributions

ZY designed the study, collected and analyzed the data, and drafted the manuscript. SW, XJ, YZ, and JL collected the data. MC and LC performed the MRI scan. FW and YT designed the study and revised the manuscript. All authors approved the final version to be submitted.

## Conflict of Interest Statement

The authors declare that the research was conducted in the absence of any commercial or financial relationships that could be construed as a potential conflict of interest.
