# Supplementary material for: Decreased Functional Connectivity in Insular Subregions in Depressive Episodes of Bipolar Disorder and Major Depressive Disorder
Source: Front Neurosci. 2018 Nov 14;12:842. doi: 10.3389/fnins.2018.00842 (PMC6246657; doi:10.3389/fnins.2018.00842)
Supplement: Supplementary file 1 [file Data_Sheet_1.docx]

Supplement material

ANCOVA results with head motion as covariate.

**
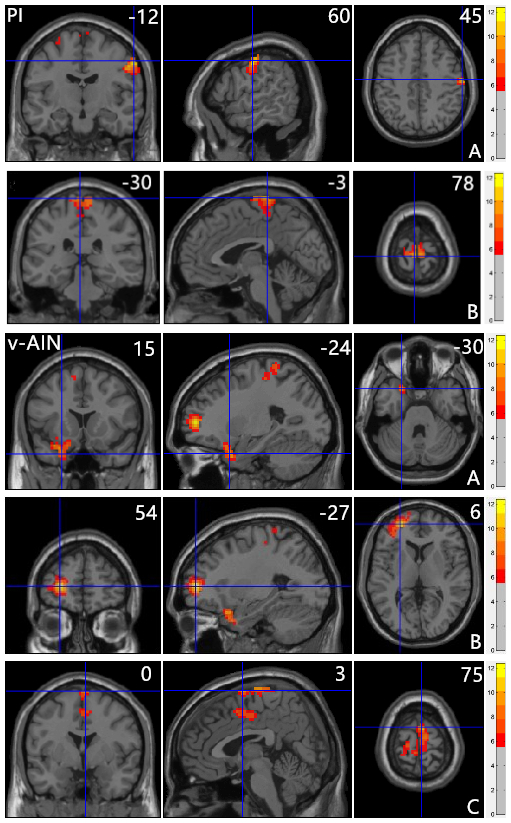
**

The significant regions of PI and v-AIN are the same with the original results without covarite.
